# Testing limits to airflow perturbation device (APD) measurements

**DOI:** 10.1186/1475-925X-7-28

**Published:** 2008-10-31

**Authors:** Erika R Lopresti, Arthur T Johnson, Frank C Koh, William H Scott, Shaya Jamshidi, Nischom K Silverman

**Affiliations:** 1 Fischell Department of Bioengineering, University of Maryland, College Park, MD 20742, USA

## Abstract

**Background:**

The Airflow Perturbation Device (APD) is a lightweight, portable device that can be used to measure total respiratory resistance as well as inhalation and exhalation resistances. There is a need to determine limits to the accuracy of APD measurements for different conditions likely to occur: leaks around the mouthpiece, use of an oronasal mask, and the addition of resistance in the respiratory system. Also, there is a need for resistance measurements in patients who are ventilated.

**Method:**

Ten subjects between the ages of 18 and 35 were tested for each station in the experiment. The first station involved testing the effects of leaks of known sizes on APD measurements. The second station tested the use of an oronasal mask used in conjunction with the APD during nose and mouth breathing. The third station tested the effects of two different resistances added in series with the APD mouthpiece. The fourth station tested the usage of a flexible ventilator tube in conjunction with the APD.

**Results:**

All leaks reduced APD resistance measurement values. Leaks represented by two 3.2 mm diameter tubes reduced measured resistance by about 10% (4.2 cmH_2_O·sec/L for control and 3.9 cm H_2_O·sec/L for the leak). This was not statistically significant. Larger leaks given by 4.8 and 6.4 mm tubes reduced measurements significantly (3.4 and 3.0 cm cmH_2_O·sec/L, respectively). Mouth resistance measured with a cardboard mouthpiece gave an APD measurement of 4.2 cm H_2_O·sec/L and mouth resistance measured with an oronasal mask was 4.5 cm H_2_O·sec/L; the two were not significantly different. Nose resistance measured with the oronasal mask was 7.6 cm H_2_O·sec/L. Adding airflow resistances of 1.12 and 2.10 cm H_2_O·sec/L to the breathing circuit between the mouth and APD yielded respiratory resistance values higher than the control by 0.7 and 2.0 cm H_2_O·sec/L. Although breathing through a 52 cm length of flexible ventilator tubing reduced the APD measurement from 4.0 cm H_2_O·sec/L for the control to 3.6 cm H_2_O·sec/L for the tube, the difference was not statistically significant.

**Conclusion:**

The APD can be adapted for use in ventilated, unconscious, and uncooperative patients with use of a ventilator tube and an oronasal mask without significantly affecting measurements. Adding a resistance in series with the APD mouthpiece has an additive effect on resistance measurements, and can be used for qualitative calibration. A leak size of at least the equivalent of two 3.2 mm diameter tubes can be tolerated without significantly affecting APD measurements.

## Introduction

Respiratory resistance is a critical measurement of lung function in a variety of respiratory disorders such as asthma, emphysema, bronchitis, pneumonia, respiratory distress syndrome, and a number of other diseases. Measurements of respiratory resistance are also useful in evaluating the reaction of the respiratory system when exposed to bronchoconstrictive or bronchodilatory drugs and airborne pollutants [1].

Whole-body plethysmography [2-4] can measure airway resistance, although the apparatus is large and non-portable. Forced oscillation [5-10] can measure total respiratory resistance, and is similar in concept to the APD.

The airflow perturbation device (APD) is a unique device for measuring respiratory resistance[1]. It is lightweight, portable, and fast, which allows for the possibility of a wide range of uses in the clinical setting. Respiratory resistance as measured by the APD is the sum of pulmonary (airway and lung tissue resistance) and chest wall resistance[1]. Unlike spirometry and plethysmography, the APD can separately measure both inhalation and exhalation resistance.

**Figure 1:**
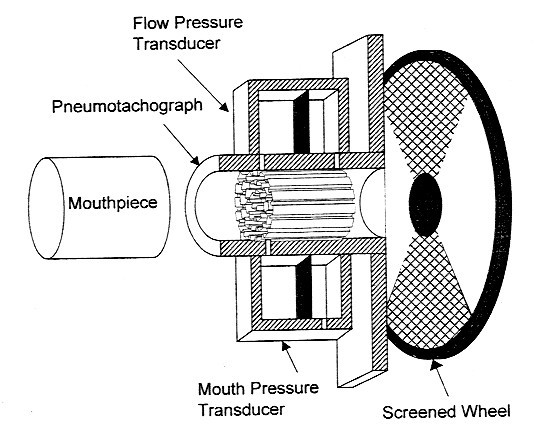
Schematic diagram of the APD showing pneumotach to measure flow rate, pressure transducer to measure mouth pressure, and the rotating wheel to perturb the airflow.

Subjects breathe normally into a disposable cylindrical mouthpiece attached to the APD (Figure 1). The air flow path from the mouth then enters the pneumotachograph and pressure transducers, where pressure and air flow are measured. A series of perturbations is created by a rotating segmented wheel that partially obstructs air flow. The depths of airflow and pressure perturbations depend on the levels of respiratory resistance and resistance of the device. Respiratory resistance can be calculated after measuring the resistance of the wheel and pneumotachograph with each perturbation. Real time measurements of respiratory resistance are displayed on the computer screen with each perturbation. At the end of 100 perturbations, average respiratory resistance, calculated by averaging inhalation and exhalation resistances, is displayed. It is necessary, with any new technology, to determine its limitations of use and its performance under non-ideal conditions. This is certainly true for technology intended for the clinical setting.

Because the APD is still being developed and is not generally available for use, important information must be obtained about its usefulness. In this respect, some speculation about possible APD uses is appropriate and then non-ideal testing conditions can be considered. It was the intent of this series of experiments to test several of these conditions to determine if further attention is warranted. If a serious problem is discovered, then additional mathematical modeling and APD software modifications may be able to address the problem. If no serious problem is found, then attention can be directed to less serious needs.

In particular, from experiences gained from using the APD, impulse oscillometry (IOS – a form of forced oscillation [11,12]), spirometry, and the body plethysmograph, four areas of concern have arisen. These were first, what effect would leaks in the seal between the lips and the mouthpiece have on measurement accuracy? A tight seal is critical for IOS accuracy, but the APD generates its signal differently from forced oscillation (which uses a mechanical pressure source). Experience has shown that achieving a seal between lips and mouthpiece can be difficult for an untrained subject. Does the APD require a tight seal, or can some leakage be tolerated while still achieving sufficient accuracy?

The second area of concern deals with the use of an oronasal mask if the APD is to be used for unconscious or uncooperative patients. Previous results [13] have shown that identical results can be expected from the APD with a mouthpiece or oronasal mask as long as breathing was exclusively through the mouth, and the mouth was kept open inside the mask. If the nostrils are not closed inside the mask, what effect does this have on APD measurements?

The third concern involves the ability to detect correctly additional resistances in the respiratory flow path. The APD could perhaps be used with respiratory protective masks as a tool to measure mask resistances while in use. Is the total resistance measured by the APD the sum of resistances of individual components, or does human respiratory resistance adjust to the imposition of external resistance by changing its value? Prior measurements in our laboratory have yielded conflicting answers to this question.

The fourth issue to be included in this study concerns the use of the APD with lengths of ventilator tubing. The most likely scenario where this situation would arise is if the APD is modified to measure respiratory resistance while the patient is assisted with a hospital ventilator. There is a particular need for accurate, quick response measurement of respiratory resistance in ventilated patients. Using feedback from the APD to control respirator function would help to prevent over-inflation of the lungs. This is especially important in the ventilation of newborn babies, whose lungs are especially sensitive and vulnerable to injury, deficient in surfactant, fluid filled, and under-supported by the chest wall [14]. Over-ventilating the lungs during this critical period of development may lead to lifelong lung disorders and even premature death. Excess inflation of the lungs in these very young patients interferes with the lungs' natural ability to secrete surfactant, a surface-active lipoprotein substance [14]. Apoptosis and proliferation caused by ventilation and resulting hyperoxia leads to pathological processes and respiratory distress syndrome in the lungs of pre-term infants [15]. Exogenous surfactant and prenatal corticosteroids are used to reduce ventilator-induced injury with success. Improved ventilator control in addition to current respiratory therapy would further diminish lung injury.

In order for the APD to be adapted for use with ventilated, uncooperative, or unconscious patients, children, or animals, different parts would need to be added or used with the device, which may or may not change the measurement values. The goals of these tests were to:

1. determine effects of leaks at the seal between the lips and mouthpiece.

2. determine if the same values can be obtained using an oronasal mask as with the standard mouthpiece. In addition, nasal resistance and combined oral and nasal resistances were measured.

3. determine if additional resistances in the flow pathway could be correctly measured.

4. see if using ventilator flexible tubing had an effect on APD measurements.

Some of the same factors investigated in this study are also concerns for the forced oscillation technique. Mouthpiece leakage and nose breathing while using an oronasal mask have been cautioned against by [9], but the magnitudes of errors incurred due to these reasons have not been given.

## Methods

The protocol for this experiment was approved by the University of Maryland Institutional Review Board. Subjects were ten healthy college students between the ages of 18 and 35. None of the subjects reported chronic respiratory problems, and subjects did not perform the test on days when they felt uncomfortably congested so that breathing was labored. Each session consisted of measurements on each of four separate APD stations. Subjects completed all four stations on ten separate days for a total of ten times, with the exception of three individuals who were not able to complete their testing. One of these three subjects completed five days of sessions, another completed seven and the third completed eight.

**Figure 2:**
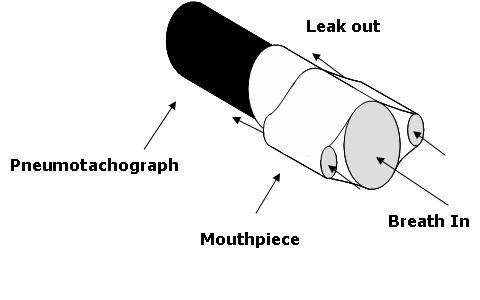
**Diagram of the mouthpiece used to test the effect of leaks around the mouth.** Small tubes on either side of the mouth provided leakage paths. This mouth piece was used in place of the regular mouthpiece with the APD.

At Station 1, subjects used four different mouthpieces. The first was the control mouthpiece, a simple cardboard cylinder that fit over the APD pneumotach. The other three mouthpieces had small, medium, and large leaks composed of a pair of 2 cm long tubes of 3.2, 4.8, and 6.4 mm (1/8, 3/16, and 1/4 inches) internal diameters respectively (Figure 2). The size of each leak was constructed to give a leak resistance to APD resistance ratio of about 1:1, 1:2, and 1:20. For each APD measurement, subjects placed their mouths securely over the mouthpiece and breathed normal breaths for approximately 1 minute with their hands holding cheeks stable to minimize vibrations. Subjects completed two measurement replications with the control mouthpiece, two measurements with the small, medium, and large leak mouthpieces, and finally two more replications with the control mouthpiece. Control replications before and after the leaky mouthpieces were made to detect changes to respiratory resistance that might have resulted from hyperventilation when compensating for the leaks.

**Figure 3:**
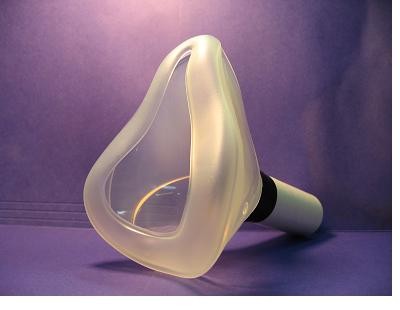
**Picture of the oronasal mask used to measure nasal, mouth, and nasal plus mouth resistances. **This mask was attached to the APD through a regular cardboard mouthpiece that fits over the pneumotach entrance.

Next, the subject moved to Station 2 where the oronasal mask was used (Adult Mask 4–5^+^; Laerdal Medical; Wappingers Falls, NY). Four measurements were taken at this station. The first measurement used the control mouthpiece. For the second measurement, the oronasal mask was placed over the mouth and nose, and the subject wore a nose clip so that breathing occurred only through the mouth. A control mouthpiece was taped to the exit of the mask so that it would fit on the mouthpiece of the APD (Figure 3). A third measurement was taken while the subject was wearing the mask, but no nose clip was worn. Finally, a measurement was taken where the subject's mouth was closed and breathing occurred only through the nose.

**Figure 4:**
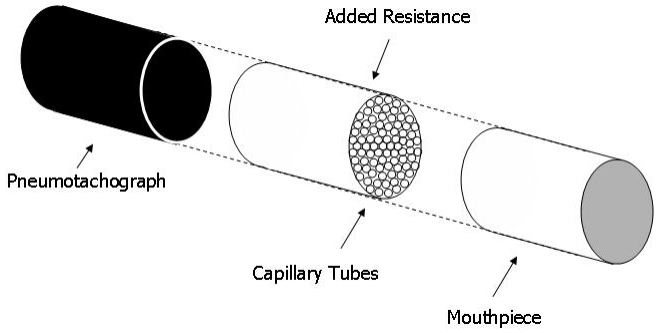
**Diagram of the additional resistance used with the APD.** This resistance was inserted between the subject’s mouth and the APD pneumotach. Constructing the resistance from capillary tubes gave laminar flow and nearly constant resistance with flow rate

At Station 3, subjects used three different mouthpieces for a total of three measurements. The first measurement was made using the control mouthpiece. The following two measurements were made using mouthpieces with an added resistance. Resistances were made using plastic tubing the same size as the control mouthpiece (Figure 4). The tubing was then filled with smaller capillary tubes with low Reynolds numbers to create additional (nearly constant) resistance over the calm breathing flow range of 0–0.5 L/sec. Control mouthpieces were taped to either end of the resistive mouthpiece. Two different sized capillary tubes were used to make two different resistive mouthpieces. The smaller capillary tubes had an inner diameter of 1.1–1.2 mm and were used to make the larger added resistance with a measured value of 2.10 cmH_2_O·sec/L. The larger capillary tubes used to make the smaller resistance had a 3 mm inner diameter and a measured resistance value of 1.12 cmH_2_O·sec/L. Both sizes were approximately 75 mm long.

Resistances of these two ensembles were determined by measuring the pressure drop across them while simultaneously measuring the flow rate through them. Pressure drop was measured with a Validyne (Cupertino, CA) DP-15 differential pressure transducer with static pressure taps in the connecting tubes just upstream and downstream of the capillary tube resistor, and flow was measured with a Fleisch #2 (Phipps and Bird; Richmond, VA) pneumotach between the flow source and the resistor. A range of steady state flows was used to determine resistance value and confirm resistance constancy.

Finally, ten different subjects with demographics similar to the previous ten subjects completed six measurements at Station 4. These measurements were completed later after it was discovered that one APD had malfunctioned. The first was a measurement using the control mouthpiece. The second was a measurement through a flexible ventilator tube 3.8 cm (1.5 inches) in diameter and 52.1 cm (20.5 inches) long. A control mouthpiece was taped onto each end of the tube. One end was connected to the APD while the subject breathed through the other end. This set of subjects completed three measurements. Statistical significance was determined using multiple paired-mean t-tests at a level of p ≤ 0.05

As a secondary feature of these tests, different APDs were used at each station in order to check on consistency between devices. It soon became clear, however, that only three of the APDs were working correctly at that time. Thus, measurements from only the first three stations were used to check APD consistency; the APD used at the fourth station was the same one used at the first station, and because the groups of subjects used at the first and fourth stations were not completely identical, differences seen between first and fourth stations could not be used to check consistency of the same device used at somewhat different times.

It was intended that each subject would visit each of four stations on each of ten days. Subjects at the first station were to be measured three times for the control condition, three times for each leak condition, and again three times for another control condition. When subjects moved to the other three stations they were to be measured once for each treatment at that station. Three modifications were made to this plan. First, a total of three measurements for each treatment at station one were found to take too much time; so, in the spirit of efficiency, the number of measurements for each treatment condition at station one was reduced to two. Second, there were subjects who could not perform on all days, so some subjects were measured for less than 10 days; one performed for five days only (see Table 6). Third, it wasn't until testing was well underway before it was discovered that the fourth APD was not operating correctly. Thus, a new cohort of subjects had to be recruited to be measured at station four for 10 days.

**Table 6:** Consistency of measurements for each subject measured with three different APDs.

Subject		1	2	3	4	5	6	7	8	9	10
Control 1 (APD 1)	Avg	2.86	5.20	3.96	5.12	4.30	3.53	4.50	4.52	2.91	4.97

	Std Dev	0.29	0.81	0.31	0.66	0.41	0.42	0.28	0.24	0.28	0.46

	No.	20	20	18	20	20	20	16	10	14	18

Control 2 (APD 2)	Avg	2.86	5.50	4.09	4.56	4.09	4.14	4.50	4.28	2.66	5.13

	Std Dev.	0.26	0.77	0.29	0.37	0.21	0.54	0.43	0.19	0.21	0.40

	No.	10	10	9	10	10	10	8	5	7	9

Control 3 (APD 3)	Avg	2.75	5.63	3.82	5.02	4.08	3.70	4.36	4.29	2.72	4.73

	Std. Dev	0.19	0.64	0.22	0.40	0.39	0.41	0.23	0.39	0.30	0.25

	No.	10	10	9	10	10	10	8	5	7	9

Resistances are given in cmH_2_O·sec/L.

## Results

Results for the four different stations are found in Tables 1, 2, 3, 4. Individual subject data entries represent the averages of ten replications taken over ten days. The column labeled "Average" is the average of individual subject data entries appearing in each row. Statistical significance at p ≤ 0.05 between paired comparisons is indicated by identical letters on each of the comparison numbers.

**Table 1:** Average results from station 1 (mouthpieces with varying leaks) from each subject.

	Subject – average over all sessions											
Station 1	1	2	3	4	5	6	7	8	9	10	Average	StDev

Control	2.86	5.20	3.96	5.12	4.30	3.53	4.50	4.52	2.91	4.97	4.19^ab^*	0.86

Small	2.82	5.00	3.68	4.62	4.08	3.22	3.95	4.28	2.68	4.30	3.86^c^	0.76

Medium	2.82	4.16	3.36	3.66	3.41	2.99	3.41	3.88	2.48	3.70	3.39^ad^	0.51

Large	2.60	3.33	2.98	3.09	2.97	2.89	3.04	3.43	2.33	3.28	2.99^bce^	0.33

Control	3.09	6.26	4.30	5.11	4.60	4.35	4.47	4.31	2.96	4.99	4.44^de^	0.95

Resistances are given in cmH_2_O·sec/L.

*Same letters indicate statistically significant differences at p ≤ 0.05.

**Table 2:** Average results from station 2 (oronasal mask) from each subject.

	Subject – average resistance over all sessions											
Station 2	1	2	3	4	5	6	7	8	9	10	Average	StDev

Control	2.86	5.50	4.09	4.56	4.09	4.14	4.50	4.26	2.66	5.13	4.18^a^	0.88

Mouth	3.58	5.62	3.91	5.58	4.43	4.49	4.82	4.44	3.83	4.48	4.52^b^	0.68

Both	3.14	5.11	3.63	5.55	4.63	4.27	4.62	3.86	5.16	4.17	4.41^c^	0.75

Nose	7.00	8.40	6.60	6.33	5.69	7.95	8.06	7.25	8.96	9.62	7.59^abc^	1.23

Resistances are given in cmH_2_O·sec/L.

*Same letters indicate statistically significant differences at p ≤ 0.05.

**Table 3:** Average results from station 3 (added resistances) from each subject.

	Subject – average resistance over all sessions											
Station 3	1	2	3	4	5	6	7	8	9	10	Average	StDev

Control	2.75	5.63	3.83	5.02	4.08	3.70	4.36	4.29	2.72	4.73	4.11^a^	0.92

Small	3.55	6.34	4.67	5.46	4.72	4.47	5.03	4.67	3.53	5.71	4.81^b^	0.88

Large	5.05	7.50	5.71	6.53	5.96	5.49	7.05	5.86	4.79	6.92	6.09^ab^	0.89

Resistances are given in cmH_2_O·sec/L.

*Same letters indicate statistically significant differences at p ≤ 0.05.

**Table 4:** Average results from station 4 (ventilator tube) from each subject.

	Subject – average resistance over all sessions											
Station 4	1	2	3	4	5	6	7	8	9	10	Average	StDev

Control	3.23	2.76	3.87	4.15	4.56	3.36	5.50	4.73	6.25	3.90	4.04	1.19

Tube	3.04	2.78	3.72	2.61	4.32	3.93	4.24	4.52	5.42	3.30	3.62	1.01

Resistances are given in cmH_2_O·sec/L.

*Same letters indicate statistically significant differences at p ≤ 0.05.

Control values in Tables 1, 2, 3, 4 are unusually high compared to previous measurements [16]. From the previous data, one would expect average respiratory resistances to fall in the range of 2.5–3.5 cmH_2_O·sec/L. Because measurements in the present study were made on the same equipment and with the same calibration as the prior measurements, it can only be surmised that high control resistances resulted from the particular set of subjects used in this study.

For station 1 (Table 1), the average resistance measurement decreased with increasing leak size. The control measurement at the beginning of the station session was lower than the control measurement at the end of station 1 for the majority of subjects, although this difference was not significant. This could be caused by the fact that after breathing into the mouthpiece with the large leak, subjects were breathing differently in compensation for the leak. Measurements taken with the large leak mouthpiece were significantly lower than measurements taken with the control and small leak mouthpieces. The medium leak mouthpiece yielded significantly smaller resistance measurements than the control mouthpiece. While the small leak mouthpiece measurements were not statistically different than control measurements, they were universally lower.

When using the oronasal mask, resistance values were slightly higher than with the control mouthpiece when subjects breathed through just their mouths and their mouths and noses, although these differences were not significant (Table 2). When subjects used just their noses to breathe, measurement values were significantly higher than the other measurement configurations.

The addition of resistances in series with the APD mouthpiece in station 3 added resistance to the entire measurement in a linear fashion (Table 3). Resistances of the additional pieces were calculated by measuring the pressure drops at various measured flow rates. The small resistance with the large capillary tubes had a measured value of 1.12 cmH_2_O·sec/L whereas it added an average of 0.7 to the control APD measurement. The larger resistance constructed from the smaller capillary tubes had a measured resistance value of 2.10 cmH_2_O·sec/L whereas it added an average of 1.98 to the control APD measurement.

The addition of a ventilator tube to the APD mouthpiece did not significantly alter the resistance values from the control mouthpiece measurements (Table 4), although the average resistance value with the tube was lower than without the tube for the majority of subjects.

APD reproducibility (Table 5) and subject consistency (Table 6) were checked by using individual subject averages for control conditions that appear in Tables 1, 2, 3. In the case of Table 1, data for only the first control condition were used. Means and standard deviations appear on the right in the first row of each Table; the slight differences in the means among different APDs were highly statistically non-significant (probability values for paired mean t tests were about p = 0.9).

**Table 5:** Reproducibility of measurements by subject for three different APDs.

Subject	1	2	3	4	5	6	7	8	9	10	Average
	
Average	2.82	5.44	3.96	4.90	4.16	3.79	4.45	4.36	2.76	4.94	4.16
	
Std Dev	0.06	0.22	0.13	0.30	0.12	0.31	0.08	0.14	0.13	0.20	0.17

Resistances are given in cmH_2_O·sec/L.

Considering the data for each subject in the control conditions, and looking at consistency of measurement for each subject measured with three different APDs, results give an average across all subjects of 4.16 cmH_2_O·sec/L with an average standard deviation of 0.17 cmH_2_O·sec/L (Table 5). Standard deviations for individual subjects were all less than 10% of the mean for that subject, and many were in the range of 5% of the mean.

## Discussion

Uncooperative patients, such as children, may not be able to provide a reliable seal around the mouthpiece of the APD. Because of this, it is necessary to see how much error can be tolerated in the seal without significantly changing the resistance measure by the APD. The smallest leak, consisting of two tubes with a 3.2 mm diameter, did not significantly change the resistance measurement in the ten subjects. However, the medium and larger leaks did. From our study, a leak of size of 4.8 mm (or about one tenth of APD resistance) on each side of the mouth would change the resistance measurement significantly. Testing on more subjects may give a definitive leak size that can be tolerated for the APD.

Most of the APD baseline resistance comes from the Fleisch #2 pneumotach (Phipps and Bird; Richmond, VA) used to measure flow rate. Resistance of the pneumotach is about 0.31 cmH_2_O·sec/L. Resistance of the wheel varies between zero and about 1 cmH_2_O·sec/L. Resistances of the leakage mouthpieces were about 0.04, 0.45, and 0.72 cmH_2_O·sec/L, calculated from the Poiseuille formula (R = 128 μL/(πd^4^) [17]); without accounting for Bernoulli effects at inlet and outlet.

When dealing with leak effects, there arises a question of sufficient accuracy of the respiratory resistance measurement. Given that the APD measures only one aspect of respiratory mechanics (respiratory resistance), other pulmonary function tests will be required in order to fully characterize the respiratory health of a patient. Nonetheless, the APD will probably be found to be a quick and easy indicator of some respiratory pathologies. The APD has been used on nearly 2000 ambulatory subjects not screened for respiratory abnormalities [16]. Respiratory resistance was tested for dependence on age, body mass, height, sex, and body mass index. The best correlation for resistance among children ages 18 or less was with age. Resistance in this group decreased hyperbolically as children grew older. The best correlation for adults ages 19–88 was gender, with females exhibiting significantly higher resistances than men. Resistances of adults tended to remain nearly constant with age, but the spread of resistances tended to increase toward higher resistances as they aged. The range of measurements on this group of people is quite large (standard deviation of about 20% of the mean for adults), and indicates that classification of a patient as abnormal probably depends more on differences between exhalation and inhalation resistances, and resistance changes, than with any absolute level of resistance. We had occasion to measure respiratory resistances of asthmatic children outpatients (unpublished observations). Some of these had expected elevated resistance values; some had normal resistances controlled by drugs; and some maintained abnormally large thoracic gas volumes to compensate for high airway resistances. Absolute levels of resistances for these patients were not found to be a good indicator of asthma severity. On the other hand, where there is a large disparity between inhalation and exhalation resistances, there may be cause for concern (exhalation resistance is normally slightly greater (within 10%) than inhalation resistance). When inhalation resistance is significantly higher than exhalation resistance, it is probably a sign of vocal chord dysfunction (VCD), or laryngeal dyskinesia [18]. We have also noticed monotonically increasing respiratory resistances with time in subjects while they breathed cool air. This may be an indication of airway hyperactivity, and a symptom of exercise-induced asthma. The point of this discussion is that there are other indicators of respiratory abnormality than absolute levels of respiratory resistance, so an accuracy standard of 10–20% is probably acceptable. A range of ± 20% encompasses a majority of the values obtained from about 2000 mostly normal adult volunteers [16], so values outside this range are the best indicators of respiratory abnormalities.

Patients with respiratory diseases will present higher resistances [16]. It might be tempting to speculate that the effect of leaks would be magnified in this case, but it is not likely to be so. Assuming that flow rates in diseased patients are equivalent to those in nondiseased patients, the effect of a leak at the mouth would be to shunt the same proportion of flow from the mouth to the outside with both normals and diseased patients. After all, the resistance from the mouth to the outside, comprising the pneumotach and wheel (APD resistance), does not change with the state of the patient.

If flow perturbations were the same for both kinds of patients, then the effect of a leak should be exactly the same for both. However, higher respiratory resistance results in lower flow perturbation magnitude, even if respiratory flow rate is maintained at the same level. The magnitudes of the flow perturbations are typically much smaller than the magnitudes of the pressure perturbations, and a leak may render the flow perturbations undetectable. Leaks, therefore, probably have a secondary effect that could be overcome with an APD design yielding a larger perturbation magnitude.

The mouth leakage devices used in this test were probably not ideal. It is difficult to characterize leaks except to say that they occur. Leaks can be quantified by size, relative parallel resistance, or flow rate. The ones used here were based roughly on parallel resistance relative to the resistance of the APD. But as the wheel rotates, APD resistance changes, so an arbitrary decision was made to base the resistance value with the wheel in the open position. The same is true for leakage flow rates compared to flow through the APD.

Only three leakages were tested. This should have been sufficient to detect significant problems in respiratory resistance measurement accuracy. As shown in Table 1, each greater leakage mouthpiece decreased the resistance measurement by approximately 0.4 cmH_2_O·L/sec compared to the previous one. Based upon the control resistance value, the leakages presented by the three leakage mouthpieces affected resistance measurement accuracy by about 10%, 20% and 30%, respectively. If a measurement accuracy of ± 10–20% is acceptable, as discussed previously, then some leakage can be tolerated when using the APD. It is not likely that a leak as large as the biggest one tested here would be able to be tolerated by a cooperative patient, because she/he would have to breathe much more heavily for the APD to acquire a flow signal to make a measurement. Our experiences from testing about 2000 children and adult subjects with the APD [16] have shown that it is small children who are the most likely to fail to make a good seal with their lips around the mouthpiece. Also, if the height of the APD is maladjusted relative to the height of the subject, then the cardboard mouthpiece that we often use can loosen from the pneumotach. Failure to obtain a resistance measurement in a reasonable amount of time leads the technician making the measurement to begin looking for leaks. If one is found, it is corrected. If one is not found, then the subject is requested to breathe somewhat more deeply. The point of this is that the most likely leaks to occur in actual practice, the small ones, will not affect APD measurement accuracy to a significant degree.

Dynamic responses of the flow and pressure transducers used in the APD could seriously affect measurement accuracy. Flow transducers used in this study were Fleisch penumotachographs, notoriously known for poor dynamic performances [19]. Smaller Fleisch pneumotachs tested in our lab have better fidelity than larger ones. The Fleisch #2 pneumotachs used for this study had cut off frequencies at greater than 10 Hz. Other flow transducers, for example the Med Graphics (St. Paul, MN) #758100-004 PreVent pilot tube flowmeter, have better dynamic performances but are inherently nonlinear.

The strain-gage pressure transducers used, DCODINDRY and DC020NDR4 (Honeywell; Oak Creek, WI) have not been tested in our lab for frequency response, but have been assumed, based upon manufacturer's specifications, to have more than adequate dynamic response for our use. This should be true even with the approximately 10 cm long flexible tubes connecting flow and pressure transducers.

Transducer dynamic response has smaller importance on APD measurements if relative rather than absolute measurements are made; that is, if differences between inhalation and exhalation resistance, or if differences of resistance from one condition to the next are used. If an absolute resistance measurement is important for a particular application where relative measurements cannot be made, then the APD should be calibrated to known resistances. This will compensate for limited dynamic response of the transducers.

The APD uses time domain analysis as compared to frequency domain analysis in forced oscillation. One disadvantage of operations in the frequency domain is the time necessary to obtain multiple samples; each data point usually requires an entire repetitive (sine) wave to be completed. At high frequencies, this time is relatively short. At low frequencies, the time can be much longer.

Time domain analysis requires just some arbitrarily small time between samples in order to obtain multiple data points, and so can be much faster in response. Time domain analysis, however, is more likely to suffer from noise effects than is frequency domain analysis, so some extra time is usually spent obtaining a sufficient number of samples to average.

Pressure and flow transducers that exhibit typical first order frequency domain roll off of -6 dB/oct will respond exponentially in the time domain. With small enough time constants, transducers will reproduce actual data samples with sufficient accuracy. Transducer time constants and accuracy specifications can limit the sampling rate in the time domain.

Different versions of the APD have used sampling rates of 200 and 500 Hz. Assuming that times equal to five time constants are required for a complete response to a sudden change of input signal, these sampling rates require transducer time constants of 1 m sec and 0.4 m sec, respectively. Corresponding cut off frequencies are about 150 and 400 Hz. The transducers used in the APD, especially the flow transducer, do not have cutoff frequencies even closely approximating either of these values. Thus, calibration of the APD against known resistance values is required for the highest accuracy possible. Even without calibration, however, laboratory checks of known resistance values, such as reported in this study, show good agreement between actual and measured values. Comparison tests have always shown differences in the right directions (increasing or decreasing).

The oronasal mask may be used in conjunction with the APD for unconscious or ventilated patients, uncooperative patients who are unable to make a tight seal around the APD mouthpiece, small children, and animals. Using this mask, nasal vs. mouth resistance was measured when subjects breathed only through one or the other. Under certain circumstances it may be useful to measure nasal resistance only. As expected, resistance increased significantly by an average of 3.07 cmH_2_O·sec/L when breathing through the nose rather than through the mouth using the oronasal mask. This disparity is to be expected because of the small diameter of the nostrils and also a more tortuous path compared to the mouth and throat.

The oronasal mask was also used to test the effect of breathing through both the mouth and the nose on respiratory resistance. It was unknown how the parallel combination of unrestricted nose/mouth breathing would affect the resistance and how carefully measurements made with the oronasal mask would have to be. Because our results did not show a significant difference in parallel nose/mouth breathing and solely mouth breathing, we conclude that either can provide an accurate measure of respiratory resistance using the oronasal mask in combination with the APD. These results confirm findings of the study by Wong and Johnson [13] where measurements made with the control mouthpiece and the oronasal mask were not found to be significantly different when tested on a subject pool of forty-seven individuals.

Use of an oronasal mask with the APD has its difficulties. The oronasal mask would be used in instances where the patient cannot or will not seal a cardboard mouthpiece with the lips. This can occur if the patient is unconscious, incapacitated in some way, or if a nasal resistance is to be measured. The oronasal mask must be pressed into the flesh of the face in order to seal it properly, but that has not seemed to be much of a problem in previous tests [13]. If the seal is critical, and holding the mask on the face cannot be easily accomplished, then the use of a sealant, such as lubricating gel, can be considered. In prior tests with pigs, we have used smooth peanut butter as a sealant; it is cheap, nontoxic, and plentiful, but somewhat difficult to clean from the faces of humans.

Less obvious is the difficulty with making a resistance measurement through the mouth inside an oronasal mask. When the lips or the teeth are not completely opened the partial obstruction of the air passage can increase the resistance measurement significantly. We have not yet dealt seriously with this matter, but some means must be developed for standardization of mouth configuration when using an oronasal mask with the APD. In the work reported in Wong and Johnson [13] when care was taken to assure the mouth was open inside the oronasal mask, there was no significant difference between resistances measured at the mouth with oronasal mask or cardboard mouthpiece.

Some of the same factors investigated in this study are also concerns for the forced oscillation technique. Mouthpiece leakage and nose breathing while using an oronasal mask have been cautioned against by Oostveen et al. [9], but the magnitudes of errors incurred due to these reasons have not been given.

Using the information in Table 2 for mouth only, nose only, and combined mouth and nose resistances allows calculation of portions of resistance appearing only in the mouth, only in the nose, and common to both resistance measurements. Mouth only resistance is in parallel with nose only resistances, and the parallel combination of mouth and nose resistances is in series with the common resistance from the throat to the chest wall. Designating m = mouth only resistance, n = nose only resistance, and c = common resistance gives three equations:

m + c = 4.5

n + c = 7.6

$$\frac{mn}{m+\text{n}}+\text{c}=\text{4}\text{.4}$$

From these the following can be calculated:

c = 3.8

m = 0.7

n = 3.8

Local nose resistance is more than five times as large as local mouth resistance and contributes an amount equal to the rest of the respiratory system to overall respiratory resistance.

Lemes and Melo [20] reported on a study using forced oscillation (FO) to measure nasal resistance of normals and nasal obstructive patients. Their subjects breathed normally into a small oronasal mask connected to their measurement device by a 48 cm long ventilator tube. Their average resistance and standard deviation for 24 normal subjects were 5.2 ± 1.2 cmH_2_O·sec/L. Values obtained in the 10 volunteers for the present study were 7.6 ± 1.2 cmH_2_O·sec/L. Although the difference in means between the two groups may be due to many causes, it is possible, based on results we obtained with the APD connected to the subject through a 52 cm long ventilator tube, that the ventilator tube they used reduced the resistance measured by Lemes and Melo [20] below the value that they would have measured if subjects had breathed into their FO device without the tube. This speculation is supported by their results that showed reduced measured resistance without cheek support compared to hands supporting the cheeks. The flexible ventilator tube can easily act as a parallel pathway for oscillations to be shunted to the atmosphere.

In addition, attention is drawn to the smaller standard deviation obtained in the present study compared to the Lemes and Melo study. Although this difference has not been formally studied in our lab, we have consistently observed that standard derivations of measurements made with the APD are smaller than the same measurements made with IOS, a form of FO. Hence, it is not surprising that the standard deviation of the present measurements is smaller than that reported by Lemes and Melo.

Especially with the current hand-held version of the APD [21], nasal resistance measurements should be very easy to make using an oronasal mask. The hand-held APD is light and very portable, and can easily be brought to the patient, wherever that may be.

Adding a resistance to the mouthpiece of the APD that is constant with variation in flow serves several purposes. First, adding resistances can be used to calibrate the APD. Second, adding a known resistance will show whether additional resistance has an additive effect or if there is a more complicated mechanism whereby the respiratory system compensates in some way for external resistance.

Additional resistances between the APD and the mouth produced higher overall resistance readings, but the differences were not the same as measured values of inserted resistors. In both cases, adding resistors resulted in lower than expected readings. The causes of these discrepancies are open to speculation. It may be that flow patterns entering and leaving the resistors were different during the APD measurement compared to the condition during which their resistances were measured by themselves. This may have resulted in different entrance and exit losses between the two measurement conditions. These discrepancies may also be as the result of some water accumulation in the recesses of the tubes, either through condensation or remaining from washing. Care was taken to thoroughly dry the resistors after each washing, but some moisture may have remained anyway. Discrepancies may also have been caused by some accommodation in the respiratory system to the added pressures and dead volumes that the resistors represented. Lastly, the differences in resistance values may be due to APD software that looks for thresholds of resistance before recognizing when a perturbation is occurring. Additional resistance can change the flow and mouth pressure existing when data analysis begins and ends. Further experimentation is necessary to distinguish among these possibilities.

Several methods have been studied to develop a technique for non-invasively monitoring respiratory resistance in ventilated patients. Among the techniques investigated are the interrupter technique, single breath occlusion, forced oscillation, and Delta-inst [22-24]. All available techniques leave elements to be desired that can possibly be fulfilled by the addition of the airflow perturbation device to the battery of lung function monitoring tools.

In a study by Snepvangers et al. [22], early neonatal respiratory resistance and compliance were assessed in preterm infants (less than 37 weeks of gestation) with no congenital abnormalities. Airway resistance was measured via single breath occlusion technique (SBT) during the first three days of life and again at 1 year of age. Increased resistance was shown to have a positive correlation with poor respiratory outcome in the first year. Thus, accurate measurement of respiratory resistance is crucial in predicting infant mortality and respiratory disease.

Babik et al. [23] studied measurements of resistance using the interrupter technique and compared it to total respiratory resistance, which was determined at the airway opening with pseudorandom oscillations of 0.2–6 Hz at end inspiration. The correlation of these two values was poor and it was concluded that the resistance measured by the interrupter technique was very insensitive to changes in lower airway resistance.

Although the APD is similar to the interrupter technique, there are crucial differences. The APD does not completely obstruct flow, as is done in the interrupter method. Therefore, the subject can still breathe if the device were to malfunction. Also, the APD makes all of its measurements at the same time, making all resistance, pressures, and volumes identical for all of the measurements [25].

In a study by Johnson and Sahota [25], APD measurements of resistance of excised sheep lungs in a respiratory chamber were compared to measurements made with the forced oscillation technique. The measurements were found to be similarly correlated to the airway resistance measured invasively. In comparing measurements made by the APD with plethysmographic resistance, regression analysis showed the relationship to be statistically significant.

Although adding a ventilator tube to the mouthpiece of the APD did not significantly change the resistance measurement, several difficulties were noted during the tube measurement. Subjects commented on feeling slightly sick, feeling like they were running out of air from hyperventilation. This was probably caused by the large amount of dead space in the tube, leading to accumulation of CO_2_, which the subjects then breathed. Subjects had to breathe for much longer to obtain 100 inhalation perturbations for this APD station, which extended the duration of discomfort. Dizziness and lightheadedness were noted more frequently on this test than any other in the study. These effects will definitely need to be taken into account if tubing is connected to the APD to make it compatible with ventilated or unconscious patients. It is also possible that CO_2 _dilates the airways. This phenomenon would confound the results of the study because both the effects of the tube and the larger airways could be acting on the resistance measurement.

The result that APD measurements with and without the ventilator tube were statistically indistinguishable means that it is likely that the APD could be modified to measure respiratory resistance of ventilated patients and produce valid measurements. The fact that results with the tube were statistically nonsignificantly lower than without the tube means that it is possible that values with a tube cannot be compared directly with values taken without a tube. Differences between the two conditions may be due to respiratory changes because of the extra dead volume that the tube represented. Subjects would have had to breathe harder with the tube and would have inhaled higher CO_2 _concentrations. Because of this result, any use of the APD with a ventilator will have to try to avoid long lengths of ventilator tubing between the APD and the patient. Among all of the techniques available, the APD appears to have great promise as an effective technique for measuring total respiratory resistance in ventilated patients and controlling the ventilator functions based on the measurements from the patient. The APD could also be adapted to accommodate small children, unconscious patients, and animals. This study was designed to assess the effects of changes to the APD measurements from adaptations to the hardware.

Data comparing individual subject measurements using three different APDs demonstrate that the APD technique is very consistent. Standard deviations for each subject are small proportions of individual means, in all cases less than 10%, and more typically less than 5%. That the APD is also sensitive to differences in resistance is shown by the range of measured respiratory resistances, from 2.76 to 5.44 cmH_2_O·sec/L. Above all, a valuable pulmonary function measurement must demonstrate consistency for the same condition and sensitivity to changes. Data in Table 5 illustrate both attributes.

Data demonstrating measurement consistency for each subject when measured by the same APD is shown in Table 6 for each of three APD devices. Standard deviations range from about 5% of the mean to about 16% of the mean. Rows marked "numbers, or (No.)" indicate the number of readings taken on that particular subject to give the mean and standard deviation. These data illustrate APD measurement reproducibility.

A different APD was intentionally used at each station so that a comparison could be made among nearly identical devices calibrated in the same ways. Some of the subjects breathing at Station 4 were not the same as subjects at the other three stations, so the proper comparison to be made uses data from Tables 1, 2, 3.

## Conclusion

A leak of up to 3.2 mm diameter on each side of the mouth caused by an inefficient mouth seal will not change measurements significantly in normal subjects. Using an oronasal mask or a ventilator tube in conjunction with the APD will not significantly change measurements (at least for the tube used in this study), but will require consideration of subject comfort due to dead volume and CO_2 _buildup. Adding a known resistance changes the APD measurement consistently, and therefore can be used to calibrate the device. This study confirmed the versatility of APD capabilities for future adaptations and possible use for a wide variety of patients.

## Competing interests

The authors declare that they have no competing interests.

## Authors' contributions

ERL conducted the bulk of the measurements for this study and wrote the first draft of this manuscript. ATJ conceived of the study and wrote much of the interpretive protions of this manuscript. FCK supported the technical and instrumentation portions of this study. WHS provided data and subject identification. SJ assisted with measurements. NKS provided specific support for the APD and its software. All authors read and approved the final manuscript.
